# The Influence of the Main Components of Tobacco Smoke, E-Cigarettes, and Air Pollutants on the Development of Glomerulonephritis

**DOI:** 10.3390/jcm15052043

**Published:** 2026-03-07

**Authors:** Magdalena Dzięgiel, Marek Misiak, Aleksandra Maciejowska, Katarzyna A. Lisowska

**Affiliations:** 1 Student Scientific Club, Department of Physiopathology, Medical University of Gdańsk, 80-211 Gdańsk, Poland; misiak.m2022@gmail.com (M.M.); amaciejowska@gumed.edu.pl (A.M.); 2 Department of Rheumatology, Clinical Immunology, Geriatrics and Internal Medicine, Medical University of Gdańsk, 80-214 Gdańsk, Poland

**Keywords:** air pollution, e-cigarette smoke, smoking, particulate matter, carbon monoxide, formaldehyde, oxidative stress, glomerulopathies, glomerulonephritis, IgA nephropathy, membranous nephropathy, minimal change disease

## Abstract

The influence of gaseous components of tobacco smoke, e-cigarettes, and air pollutants on the development of glomerulonephritis has been the subject of numerous studies in recent years. Glomerulonephritis (GN) often leads to progressive kidney damage and chronic kidney disease (CKD), which is a global health problem. Genetic and autoimmune factors have been shown to contribute to their development. Yet, increasing attention is being given to environmental and lifestyle-related risk factors. This paper summarizes how specific substances found in tobacco smoke, e-cigarette smoke, and air pollutants contribute to the development and progression of GN. Particular emphasis is placed on substances such as formaldehyde, heavy metals, and particulate matter, which have been shown to trigger oxidative stress, immune dysregulation, and endothelial dysfunction. A clear understanding of the contributions of those agents to kidney inflammation is crucial for developing preventive strategies and improving public health awareness. We also highlight gaps in current research and suggest directions for future investigation. Understanding consequences of cigarette smoking should be promoted to encourage people to reduce their exposure to cigarette smoke, which could prevent many diseases.

## 1. Introduction

Air pollution today is a global problem, lowering not only the quality of life but also human health [1]. Government and non-governmental organizations work on solutions to reduce air pollution. The World Health Organization (WHO) has issued guidelines stating that PM_2.5_ (particulate matter 2.5) and PM_10_ should be less than 8% and 36%, respectively. Current European Union (EU) limits stand at 99% and 96% [1]. As shown, achievable air pollution levels exceed those considered harmful to health. For example, in Poland, the mean PM_2.5_ for the whole country between 2000 and 2024 was 21.2 µg/m^3^ (recommended mean 15 µg/m^3^ per year [2]); PM_10_—30.4 µg/m^3^ (recommended mean 45 µg/m^3^ per year [2,3]). Mean cadmium (Cd) was 0.6 ng/m^3^ (with recommended mean 0.005 µg/m^3^ per year [2]), and average lead level (Pb) was 0.025 µg/m^3^ (suggested mean 0.5 µg/m^3^ per year [2]). No clear leads regarding safe exposure concentrations of nickel were registered [4]. According to the EU, children’s products are required to release no more than 0.2 mg of nickel per square centimeter per week [4]. In comparison, data for 2025 place Poland as a country with rather medium–low-level (10 ng/m^3^) PM_2.5_ and PM_10_-polluted air, compared to the Scandinavian Peninsula (less than 5–10 ng/m^3^) [5]. The highest levels supervene in India (40 ng/m^3^) and Algeria (80 ng/m^3^) [5]. Moreover, the level of polycyclic aromatic hydrocarbon (PAH) air concentration in Europe decreased from 100% in 2005 to 70% in 2020 [1].

Reports from the European Environment Agency (EEA) in 2025 indicated that lead concentrations exceeded the EU annual limit at two sites in Hungary. In the remaining 27 EU countries (excluding Malta), Norway, Serbia, and Switzerland, lead concentrations were below the cut-off value. Moreover, in all 27 EU countries, Norway, Serbia, and Switzerland (a total of 30 countries), Cd concentrations were reported to exceed the annual target value [1].

Regarding other components of air pollution and cigarettes/e-cigarettes, the Environmental Health Criteria [6] states that, within 90 min, there is a hazardous impact on the airways at concentrations ≥ 0.5 mg/m^3^ [6]. Formaldehyde is carcinogenic and acrid in every amount, but data from research show that inhalation of 0.09 mg/m^3^ formaldehyde for 30 min via oral breathing, followed by dust mite exposure, leads to a bronchial reaction at a lower allergen concentration than in background air containing 0.03 mg/m^3^ formaldehyde [7]. This timely topic can be followed daily, for example, using applications such as the European Air Quality Index & App, which is recommended by WHO [1].

Around 2000 years ago, tobacco began to be chewed and smoked as a part of cultural and religious events [8]. The earliest cigarette-manufacturing machines could produce approximately 200 cigarettes per minute, whereas modern equipment can produce around 9000 per minute. By the 1700s, smoking had become more widespread, and the tobacco industry had developed [8]. Globally, around half of the adults with asthma are current or former cigarette smokers [9]. As the WHO data indicates, smoking trends by age changed between 2000 and 2022. In 2000, 20.5% of adolescents aged 15 to 24 smoked [6]. By 2022, this percentage had decreased to 13.3% [6]. People aged 75–84 years also showed a downward trend, from 27.7% to 16.9%, respectively. During the studies, men were more often smokers than women [6]. Regionally, the highest percentage of smokers was in South-East Asia in all time ranges, where the least smoking region was Africa [6]. As for 2024, Eurostat estimated that 19.7 per cent of the EU population smoked daily. The highest percentages were observed in Bulgaria and Turkey, and the lowest in countries in northern Europe [10].

E-cigarettes (ECs) came into widespread use around 2007. ECs are considered to be less toxic per puff compared with combustible cigarettes, but so far, their long-term effect on the human body remains a mystery [11]. In contrast to classic cigarettes, Romania, Turkey, and Spain are among the least smoking regions, whereas Poland (4.6), Iceland (4.1), and France (3.4) are major e-cigarette markets, exceeding the European mean (1.7) by more than 2-fold. When it comes to age, it is alarming that e-cigarettes are mostly used by adolescents [12]. Globally, in the age frame 13–15 years old, 6.8% of girls smoke, while in the European Region of the WHO, 10.1% of girls do. Data on boys in the same age group is more stable: worldwide, 12.5% of boys smoke; in Europe, 11.5% [12].

The detrimental impact of smoking on human health is widely recognized; however, it may be more striking to note that approximately 50% of smokers die prematurely, on average 14 years earlier than non-smokers. Furthermore, according to the International Agency for Research on Cancer, tobacco use accounts for more than 750,000 cancer cases annually in Europe that could potentially be avoided [10].

## 2. Outline of Cigarette Smoke and Air Pollution Ingredients

### 2.1. Tobacco Smoke

Tobacco smoke is a highly complex, dynamic aerosol composed of submicron liquid particles suspended in a gas phase rich in nitrogen (N_2_), oxygen (O_2_), carbon monoxide (CO), and carbon dioxide (CO_2_). Each particle constitutes a multicomponent matrix of chemical compounds generated by the distillation, pyrolysis, and combustion of tobacco and its additives. With conventional cigarettes, the burning cone reaches temperatures of >900 °C, resulting in the production of more than 7000 toxicants, with at least 250 of which are considered harmful to humans, and significant levels of highly reactive free radicals [13]. The smoke can be conventionally divided into two main phases: a gas phase, which accounts for approximately 85% of the smoke, and a particulate or tar phase, which accounts for roughly 15% [14]. This separation is typically achieved using a Cambridge glass-fiber filter, which traps particles larger than ~0.1 μm (tar phase) and allows smaller particles to pass through (gas phase) [15,16]. Broadly speaking, the gas phase mainly comprises low-molecular-weight compounds (typically below 60 Da), whereas the particulate phase contains heavier molecules with molecular weights generally exceeding 200 Da [17]. It should be noted that this biphasic classification is a conceptual simplification, as certain compounds (e.g., formaldehyde, hydrogen cyanide) may be distributed between the gas and particulate phases or partition dynamically between them during smoke dispersion [18]. Chemical compounds in both phases contribute to the overall toxicity and pathogenic potential of cigarette smoke. Nevertheless, they affect multiple organ systems, including the kidneys, in different ways, depending on their distinct distributions and mechanisms of action [19].

The gas phase is composed predominantly of CO (average concentration 20–35 mg per cigarette), CO_2_, O_2_, N_2_, and methane. This phase also contains various volatile organic compounds (VOCs), including formaldehyde, acetaldehyde, acrolein, acetone, methanol, 1,3-butadiene, benzene, and toluene, as well as other carbonyl compounds. Additional toxic constituents present in the gas phase include hydrogen cyanide (HCN), nitrogen oxides (NO_x_), nitric acid, ammonia, hydrogen sulfide (H_2_S), a broad range of hydrocarbons, gas phase nitrosamines, and heterocyclic compounds such as pyridine, pyrrole, and furans [14,20]. Unlike substances bound within the tar phase, gas-phase compounds can traverse the airway epithelial barrier and enter the systemic circulation via the pulmonary vasculature [19].

The particulate phase, commonly referred to as the tar phase, consists of liquid aerosol droplets containing nicotine (the primary alkaloid, typically 1–2 mg per cigarette), polycyclic aromatic hydrocarbons (PAHs) such as benzo[a]pyrene, tobacco-specific nitrosamines (TSNAs) including N-nitrosonornicotine (NNN) and nicotine-derived nitrosamine ketone (NNK), aromatic amines, and phenolic compounds like catechol and hydroquinone, as well as carboxylic acids, terpenoids, paraffin waxes, humectants, water, and heavy metals such as cadmium, lead, and arsenic. Additionally, this phase contains tar particles (approximately 10–15 mg per cigarette) and highly reactive free radicals. Notably, components of the tar phase are electrically charged semi-liquid particles with diameters typically between 0.1 and 1 µm (averaging around 0.2 µm), which rapidly increase in size via coagulation [21]. Consequently, they tend to deposit locally within the respiratory tract, producing predominantly local toxicity in organs such as the lungs, tongue, and pharynx, rather than exerting systemic effects [19].

### 2.2. Types of Smoke

**Figure 1:**
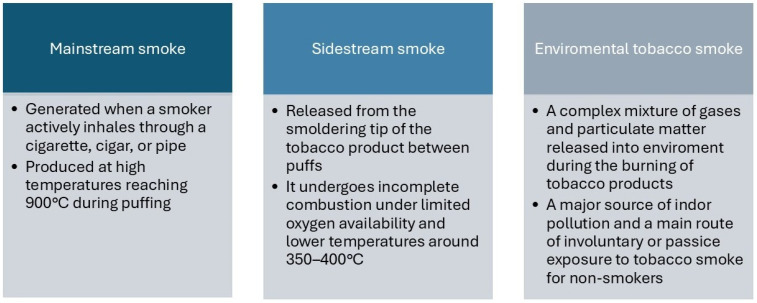
Differences between the three types of smoke.

Cigarette smoke can be classified into three forms with distinct physicochemical properties, based on variations in combustion temperature and oxygen availability [17]. Mainstream smoke (MSS) is generated when a smoker actively inhales through a cigarette, cigar, or pipe [22]. It is produced at high temperatures, reaching approximately 900 °C [23] during puffing, thereby promoting more complete combustion (Figure 1). Sidestream smoke (SSS), in contrast, is released from the smoldering tip of the tobacco product between puffs [22]. Under conditions of limited oxygen availability and lower temperatures around 350–400 °C, it undergoes incomplete combustion.

The third type, environmental tobacco smoke (ETS), is a mixture of gases and particulate matter released into the environment during the burning of tobacco products and primarily consists of SSS (85%) and a small portion of exhaled MSS (15%) from the smoker [24]. ETS is a major source of indoor air pollution and represents the main route of involuntary or passive exposure to tobacco smoke for non-smokers. [25] Additionally, certain volatile components, such as CO, may diffuse directly through the cigarette paper and further contribute to ETS even in the absence of active smoking [22].

Notably, there are differences in particle size and toxicant concentration between the smoke types mentioned. As a result of incomplete combustion, SSS contains markedly higher concentrations of many harmful compounds, including VOCs and gases. The concentration of ammonia in SSS may be up to 170-fold higher, while volatile N-nitrosamines (such as N-nitrosodimethylamine) can show SSS/MSS ratios ranging from 20 to 100. Furthermore, the particulate phase of SSS is enriched in tobacco-specific nitrosamines (TSNAs), including N′-nitrosonornicotine, with concentrations sometimes up to fourfold higher than in MSS. Elevated levels of aromatic amines (e.g., aniline, toluidine), with SSS/MSS ratios reaching 30-fold and higher, and higher concentrations of nitrogen oxides (NO_x_, SSS/MSS ≈ 4–10) further contribute to its toxicological significance [22]. Additionally, SSS exhibits a higher pH than MSS, increasing the proportion of free, unprotonated nicotine in the vapor phase, thereby enhancing volatility and bioavailability [22,26].

In addition to its distinct chemical composition, SSS differs from MSS in its physical properties, further increasing its harmful potential. The aerosol particles generated in SSS are initially smaller than those in MSS; however, after release into the environment, they tend to aggregate (coagulate) over several minutes. Despite the small primary particle size, which would typically allow deeper penetration into the alveolar regions, the tobacco smoke aerosol behaves aerodynamically as a diffuse cloud with an effective particle size of approximately 6–7 μm. This results in substantial deposition (~95%) in the upper and central airways rather than exclusively in the lung periphery. This phenomenon helps explain the observed predominance of bronchial cancers in smokers, rather than alveolar tumors, despite the fine particulate nature of tobacco smoke [17]. Moreover, children exposed to SSS are at particularly high risk, since they have smaller airways and higher minute ventilation per kilogram of body weight, which increases both the dose and the depth of particulate deposition relative to adults. Thus, the combination of higher concentrations of many carcinogens and irritants, together with the physical characteristics of the SSS aerosol, makes it a major contributor to the health hazards associated with ETS [17].

This disparity in toxicant profiles underscores the differential health risks posed by direct smoking versus passive exposure. Whereas MSS is the primary source of toxicant intake for smokers themselves, SSS constitutes the major component of secondhand smoke inhaled by nonsmokers in proximity [27]. Additionally, cigarette design elements—as filter presence, tobacco blend, rod density, and paper porosity—alongside smoking behaviors (e.g., puff volume and interval) further modulate the relative yields and composition of mainstream and sidestream smoke emissions [26,28].

These factors collectively influence the exposure levels and toxicity profiles relevant to both active and passive smoking scenarios. It is important to note that chemical and physical changes occurring after smoke exits the cigarette, as well as differences in analytical methods, may contribute to variability in reported constituent levels.

### 2.3. Air Pollution

Air pollutants are defined as chemical, physical, or biological substances, including solid and liquid particles and certain gases, present in the atmosphere at concentrations and durations that may be harmful to humans, organisms, or the environment [29]. It is important to note that some of these substances occur naturally in the atmosphere, and only their elevated concentrations have adverse effects [30,31].

Air pollution can be broadly classified by various criteria, including origin (natural or anthropogenic), location (outdoor/ambient or indoor/household), chemical composition (gaseous, particulate, or biological), and formation process (primary vs. secondary pollutants).

### 2.4. Comparison of the Components of Cigarette Smoke, E-Cigarettes, and Air Pollution

One of the fundamental factors of human health is the environment. Tobacco smoke is a complex mixture of numerous substances that pose health risks to both active and passive smokers. Of the more than 7000 identified constituents, over 100 are recognized as hazardous, and at least 69 have been classified as carcinogenic [32].

The particulate matter (PM) refers to a mixture of extremely small solid and liquid particles suspended in the air [33]. The major components of PM include soot, metals, organic compounds, sulfates, ammonium, nitrates, and other ions [34]. PM is typically categorized according to its aerodynamic equivalent diameter (AED) as coarse (PM_10_, <10 μm), fine (PM_2.5_, <2.5 μm), ultrafine (PM_0.1_, ≤0.1 μm), and quasi-ultrafine (PM_0.3_) [35,36,37]. AED is the primary factor influencing PM effects, as it determines particle deposition in the respiratory system; smaller particles can reach the alveoli, enter the bloodstream, and affect distant organs, including the kidneys. The majority of PM in the atmosphere arises from chemical interactions among air pollutants [37].

Environmental exposure is associated with increased risk of all-cause mortality and a wide range of specific diseases, representing a key environmental trigger for premature death globally [38]. Three major sources of chemical exposure—tobacco smoke, e-cigarette aerosol, and ambient air pollution—contain overlapping toxic compounds.

Due to the small size of many particles, these substances can penetrate the lungs and enter the bloodstream, thereby affecting nearly every organ in the body through mechanisms such as oxidative stress, endothelial dysfunction, and immune dysregulation [39]. Indeed, strong epidemiological evidence demonstrates that environmental pollution contributes to a broad spectrum of diseases, including various types of cancers, as well as cardiovascular and respiratory disorders [40]. In contrast, the effect on the kidneys has received comparatively less attention, despite their high blood flow, filtration capacity, and role in excreting xenobiotics, which makes those organs potentially vulnerable to circulating pollutants [41]. This vulnerability is supported by emerging experimental studies demonstrating renal susceptibility to inhaled pollutants [42]. Recent studies suggest that exposure to certain air pollutants may be associated with several kidney diseases, including chronic kidney disease and renal cancer [43,44,45,46], highlighting the need to further investigate renal toxicity as part of the systemic impacts of air pollution. According to a meta-analysis by Okoye et al. [45], individuals from communities exposed to air pollutants had lower eGFR, higher serum creatinine, and an increased risk of CKD than those unexposed. Chen et al. [46] in their review cited numerous studies showing relationships between PM_2.5_, PM_10_, SO_2_, NO_2_, CO, O_3_, and kidney function parameters. Zare Sakhvidi et al. [43] reported that several cohort studies have shown associations between kidney cancer and air pollutants, particularly NO_X_, NO_2_, and O_3_. Dahman et al. [44] demonstrated that a 10 μg/m^3^ increase in PM_10_ and NO_2_ levels is associated with a higher risk of kidney cancer.

Understanding the chemical composition, physicochemical properties, and systemic distribution of these exposures is therefore essential to elucidate their contribution to the development of glomerulonephritis. This framework also emphasizes the distinction between direct exposure, such as active smoking or vaping, and indirect exposure, such as passive inhalation of environmental tobacco smoke or ambient air pollution, both of which may deliver biologically active compounds to the renal microvasculature.

Cigarette smoke exposure exerts numerous deleterious effects on the vascular endothelium [47]. Many of these alterations arise from oxidative stress triggered by reactive oxygen species (ROS), reactive nitrogen species (RNS), and other oxidants present in smoke. Ultimately, ROS contribute to endothelial dysfunction by decreasing nitric oxide bioavailability, promoting oxidative imbalance and enhancing the expression of proinflammatory cytokines [48]. Those mechanisms were further elaborated by Dzięgiel et al. [49] in their considerations of nicotinism and glomerulopathies.

## 3. Outline of Primary Glomerulopathies

Tobacco exposure is widely acknowledged as a contributor to the progression of kidney disease. Individuals with proteinuric glomerulopathies (GN) face an elevated risk of cardiovascular morbidity and mortality. In both adult and pediatric populations with proteinuric GN, smoking is linked to a higher likelihood of developing chronic kidney disease (CKD) and to poorer renal outcomes [50]. Although the long-term renal consequences of e-cigarette use are not yet well defined, emerging evidence indicates that vaping is independently associated with CKD in a dose-dependent fashion, particularly among individuals without diabetes. These findings suggest that e-cigarette use may represent a modifiable risk factor warranting focused public health strategies [51]. It has also been reported that e-vapor exposure may increase oxidative stress and damage mitochondrial oxidative phosphorylation complexes and DNA, without a significant effect on fibrotic markers. Yet, the combination of nicotine e-vapor and high-fat diet (HFD) can increase inflammatory responses, oxidative stress-induced DNA injury, and pro-fibrotic markers, suggesting accelerated development of renal pathology. It has also been found that nicotine-free e-vapor exposure and HFD consumption suppress mitochondrial oxidative phosphorylation (OXPHOS) complex production and the deposition of extracellular matrix (ECM) proteins. This may cause structural instability, potentially disrupt normal kidney function, and further increase susceptibility to kidney disease [52]. Environmental pollution also significantly impacts global disease burden. Short-term exposure to air pollution is said to increase the risk of kidney disease-related events such as hospital admissions or death. Long-term exposure might lead to chronic systemic inflammation and oxidative stress, contributing to the development of kidney diseases. Air pollution may also exacerbate traditional kidney disease risk factors such as hypertension and diabetes. Environmental health policies are crucial for preventing and improving kidney health worldwide [53].

Primary glomerular diseases (PGD) represent the third leading cause of kidney failure globally, following diabetes and hypertension [54], and are a major contributor to end-stage renal disease (ESRD) among young adults. PGDs comprise a diverse spectrum of disorders with distinct histopathological and clinical features. The most prevalent forms include IgA nephropathy (IgAN), focal segmental glomerulosclerosis (FSGS), membranous nephropathy (MN), and minimal change disease (MCD).

### 3.1. IgA Nephropathy

IgA nephropathy (IgAN), a leading form of primary glomerulonephritis, is defined by mesangial accumulation of galactose-deficient IgA1 (Gd-IgA1) and is frequently associated with mucosal immune dysregulation and an altered Th1/Th2 balance [49]. Beyond intrinsic immune mechanisms, environmental exposures appear to influence disease progression. Air pollution, particularly PM_2.5_, has been associated with the progression of chronic kidney disease in the general population and may independently increase the risk of kidney failure in patients with IgAN. However, broader validation across different geographic and demographic settings remains necessary [55]. Tobacco exposure represents another modifiable environmental factor. Evidence from Cha et al. [56] indicates that smoking contributes to dose-dependent renal function decline and hypertension, potentially through microvascular damage. Consistently, Yamamoto et al. [57] identified cigarette smoking as a significant dose-related prognostic factor in IgAN and advocated smoking cessation as part of comprehensive therapeutic management.

### 3.2. Focal Segmental Glomerulosclerosis

Primary FSGS is a relatively rare, immune-driven form of glomerulonephritis and accounts for 16.6% of FSGS cases identified in renal biopsy series from diverse populations. The standardized incidence between 2010 and 2021 was estimated at 1.7 cases per 100,000 patient-years [58]. Rather than representing a single disorder, FSGS reflects a morphological pattern of injury characterized by focal and segmental glomerular sclerosis, typically resulting from podocyte damage. Histopathological assessment remains the cornerstone of diagnosis, while clinical features—including proteinuria severity and etiologic context—guide patient stratification. The condition can occur at any age and frequently progresses to ESRD [58]. Accumulating evidence highlights the pivotal contribution of inflammatory and immunologic pathways to glomerular injury. Podocytes, traditionally viewed as structural components of the filtration barrier, exhibit immune-like properties and may influence both innate and adaptive immune responses. These insights have expanded the understanding of disease mechanisms and opened new perspectives for targeted therapeutic interventions [59].

### 3.3. Membranous Nephropathy

Membranous nephropathy (MN) is a non-inflammatory autoimmune disorder affecting the glomeruli of the kidney. It is characterized by thickening of the glomerular basement membrane (GBM), typically resulting from immune complex deposition, and commonly presents with nephrotic syndrome. The disease accounts for approximately 30% of cases of ESRD [60]. It is characterized by complement-mediated proteinuria and an increased risk of renal failure. Few antigens, such as neutral endopeptidase, phospholipase A2 receptor (PLA2R), thrombospondin domain-containing 7A (THSD7A), that arise in adulthood, and the characterization of antibody-binding domains (epitopes) have been discovered so far. Some evidence is emerging that protein kinase C-binding protein NELL-1 is associated with 5–10% of PMN cases that are PLA2R- and THSD7A-negative. Exostosin 1 (EXT1), EXT2, NELL-1, and contactin 1 (CNTN1) are also suggested to be associated with MN [60]. Several interacting loci in human leukocyte antigen (HLA)-DQ, HLA-DR, and PLA2R1, as well as classical HLA-D alleles, have been identified as risk factors, with associations varying by ethnicity [61]. There are also reports of an association between HLA-DR3 and membranous nephropathy [62]. C3 glomerulopathy, due to its membrane-attacking complexes, may also be considered a type of membranous glomerulopathy, in which the etiology begins with dysregulated activation of the basal C3 protein [63]. Activated C3 enhances the alternative complement pathway via a positive feedback loop, promotes recruitment of inflammatory cells, and contributes to the formation of the membrane attack complex, ultimately leading to renal injury. Membranous nephropathy (MN) represents the leading cause of nephrotic syndrome in Caucasian adults, accounting for approximately 30% of cases, with an annual incidence of 1.7 per 100,000 individuals [64,65]. The disease shows a clear male predominance (male-to-female ratio of 2:1) and occurs most frequently between ages 50 and 60. Zhu et al. [66] reported a causal relationship between nitrogen oxides and the risk of MN. The study found that nitrogen oxides in the air are a risk factor for MN, increasing the risk of the disease and accelerating its progression 194. Other authors, like Yamaguchi et al. [67], say that smoking is a significant and dose-dependent risk factor for idiopathic membranous nephropathy (IMN) progression, which brings the conclusion that all patients with IMN who smoke should be encouraged to quit.

### 3.4. Minimal Change Disease

Minimal change disease (MCD) is one of the leading causes of idiopathic nephrotic syndrome (NS). It is marked by severe proteinuria, which results in edema and reduced intravascular volume, accounting for approximately 15% of adult patients with idiopathic NS and reaching up to 70–90% in children > 1 year of age. Recently, the use of anti-CD20 antibodies has led to long-term remission [68]. In MCD, the glomeruli appear normal on light microscopy but still show electron-microscopic changes that lead to significant proteinuria. Recent progress in whole-exome sequencing reveals pathogenic mutations in familial cases of steroid-sensitive nephrotic syndrome (SSNS). In most cases, circulating permeability factors have been implicated alongside T-cell dysfunction. Relapse prediction, disease activity, and steroid response are crucial [69]. Understanding the pathogenesis of MCD remains challenging due to the complex crosstalk among immune mechanisms, glomerular cellular components, and genetic factors. Additional difficulties arise from the heterogeneous nature of the disease and the lack of reliable experimental animal models [70].

## 4. Proposed Mechanisms of Action of Substances Contained in Air Pollution, Cigarettes, and E-Cigarettes

### 4.1. The Effects of PM_2.5_

#### 4.1.1. Influence of PM_2.5_ on IgA Nephropathy

Numerous studies show that PM_2.5_ impairs kidney function, leading to a decline in estimated glomerular filtration rate (eGFR) with higher PM_2.5_ concentrations. Polycyclic aromatic hydrocarbons (PAHs) are one of the main components of PM_2.5_. Because PM_2.5_ is classified as carcinogenic, mutagenic, and teratogenic, this classification provides insight into its harmful effects on podocytes [71]. Moreover, benzo[b]fluoranthene, the PAH with the highest concentration, was shown to correlate with decreased synaptopodin-actin binding, a renoprotective protein that primarily affects podocytes [71]. Its role is to bind to α-actinin-4 and modulate its actin-bundling activity. Synaptopodin promotes stress fiber formation by stabilizing the GTPase RhoA, while inhibiting filopodia by disrupting Cdc42-IRSp53-Mena signaling complexes [72].

Luo et al. [73] reported that individuals with biopsy-confirmed IgA nephropathy (IgAN) who had been exposed to elevated PM_2.5_ levels prior to study enrollment exhibited greater 24-h urinary protein excretion and higher hemoglobin concentrations. Furthermore, each 10 μg/m^3^ increase in the mean annual PM_2.5_ level before enrollment was associated with a 1.14-fold higher risk of progression to ESRD. Some evidence suggests that PM_2.5_ can shift the Th1/Th2 balance towards Th2 [74], which is associated with Gd-IgA1 production by B cells and IgAN progression [75].

According to the stratified analysis, patients exposed to lower satellite-derived PM_2.5_ aerosol optical depth had an ESRD risk comparable to that observed in individuals with elevated Gd-IgA1 levels. Conversely, those with both higher PM_2.5_ exposure and increased serum Gd-IgA1 concentrations demonstrated a marginally higher risk of ESRD compared with patients presenting lower Gd-IgA1 levels [73]. In contrast, in a subgroup analysis of IgAN patients with measured Gd-IgA1 levels, the authors reported no significant interaction between PM_2.5_ exposure and serum Gd-IgA1 concentrations.

#### 4.1.2. Influence of PM_2.5_ on Membranous Nephropathy

PM_2.5_ has been linked to air pollution-associated membranous nephropathy in a Chinese study [76]. The frequency of MN was higher in the northern region, the most polluted area in China. Additionally, in this study, most of the MN were PLA2R-related.

Experimental animal models have demonstrated that PM_2.5_ exposure enhances autoantibody production and promotes immune complex formation [76]. This effect may be mediated by cytokines released in the airways in response to air pollution, which can enter the systemic circulation and modulate autoimmune processes at distant sites. In vivo studies directly on the lungs demonstrated induced expression of the oxidative stress marker gene heme oxygenase 1 and increased levels of inflammatory cytokines, neutrophils, and activated macrophages in bronchoalveolar lavage fluid after exposure to PM_2.5_ at unaltered concentrations via the VACES aerosol enrichment system [77]. Air pollution also increases circulating levels of inflammation mediators, including TNF-α, IL-6, and plasminogen activator inhibitor (PAI), and genetic polymorphisms in these cytokines are associated with the development of MN [76].

A study by Zhu et al. [66] examined PM_2.5_, PM_2.5–10_, and PM_10_ (as well as nitrogen dioxide and nitrogen oxides) as genetically predicted factors in deoxyribonucleic acid methylation and found that they did not contribute significantly to increased MN risk. However, mitochondrial DNA methylation may represent an upstream event in mitochondrial dysfunction, contributing to elevated oxidative stress and ultimately leading to renal tubular epithelial cell injury and apoptosis [78]. These findings contradict other data presented, raising questions about what may be driving the differences between those studies and leading to such disparate outcomes.

As is well known, MN is primarily caused by circulating autoantibodies against PLA2R and THSD7A. Studies show that PM_2.5_ exposure can induce PLA2R expression outside the kidney, thereby increasing the incidence of MN [79]. Recent studies have highlighted the role of the lungs in MN pathophysiology [80]. Zhang et al. [81] demonstrated that PLA2R is expressed in human alveolar epithelial cells. Moreover, podocytes exposed to supernatants from bronchial epithelial cell cultures treated with PM_2.5_ showed increased PLA2R expression and reduced nephrin expression, indicating podocyte injury. In healthy kidneys, PLA2R is faintly expressed in podocytes, whereas in MN kidneys its expression is significantly increased, contributing to the production of anti-PLA2R antibodies [82].

Another mechanism associated with lung exposure to air pollution is inflammation. Both fly ash PM and standard PM_2.5_ induce pulmonary inflammation, resulting in increased neutrophil counts in perivascular areas and activation of alveolar macrophages [83]. Acute PM_2.5_ exposure typically favors the activation of proinflammatory M1 macrophages, whereas chronic exposure promotes the activation of anti-inflammatory M2 macrophages. Both macrophage types amplify type 2 immune responses by interacting with eosinophils, Th2 cells, and epithelial-derived cytokines [84]. Since activated neutrophils and macrophages release neutrophil extracellular traps (NETs) and macrophage extracellular traps (METs), respectively, Zhang et al. [81] hypothesize that PLA2R expressed on these cells can be released into the inflammatory space when NETs and METs are released. Indeed, studies confirm that PLA2R is present in both macrophages [85] and neutrophils [86].

Inflammation enhances the immunogenicity of autoantigens and affects the antigen-processing capacity of antigen-presenting cells (APCs), thereby contributing to the autoimmune response. It is speculated that the PLA2R antigen may be captured by mature APCs, thereby increasing its accessibility for anti-PLA2R antibody production [81]. Ke et al. [87] reported that treatment of podocytes with different active protein C (aPC) concentrations led to the conclusion that aPC can increase the phosphorylation of ERK1/2, promote the translocation of Y box binding protein 1 (YB-1) to the nucleus, and reduce the expression of PLA2R1, which results in inhibition of cell apoptosis. Thus, aPC can improve membranous nephropathy by affecting podocyte apoptosis through the ERK1/2/YB-1/PLA2R1 axis [87].

In situ immune complexes (ICs) are formed when circulating anti-PLA2R antibodies bind to endogenous PLA2R expressed within the glomeruli. PM_2.5_ exposure may also induce renal injury and modify the local microenvironment, potentially altering the molecular structure of the PLA2R antigen on podocytes and influencing the binding affinity of anti-PLA2R antibodies [88]. Furthermore, evidence indicates that PM_2.5_ dysregulates immune processing by promoting antigen presentation and strengthening autoimmune responses. Experimental data suggest that air pollution and oxidative stress drive the maturation of antigen-presenting cells (APCs), enabling the formation of antigen peptide–MHC complexes necessary for T cell receptor (TCR) activation [81].

There is a positive correlation between anti-PLA2R antibodies and oxidative stress markers, including malondialdehyde (MDA). Chronic exposure to PM_2.5_ is well recognized as a trigger of oxidative stress, which plays a major role in pulmonary epithelial injury. In an experimental study evaluating the effect of PM_2.5_ on PLA2R expression in bronchial epithelial cells, significant upregulation of PLA2R and increased oxidative stress were observed in Beas-2B cells [81]. Furthermore, the overexpression of PLA2R and oxidative stress markers was reduced primarily by the antioxidant glutathione (GSH), indicating ongoing oxidative stress. The adverse effects of PM_2.5_ on renal function involve multiple mechanisms, including inflammation, oxidative stress, cell apoptosis, DNA damage, and autophagy. To explore the influence of PM_2.5_ exposure on MN development, supernatants from bronchial epithelial cells exposed to PM_2.5_ were used to treat podocytes. Findings reveal heightened PLA2R expression and podocyte injury, highlighting the potential interplay between the lung and kidney in response to PM_2.5_ exposure [88].

### 4.2. The Effects of Carbon Monoxide

Carbon monoxide (CO) is a poisonous, colorless, odorless, tasteless, nonirritating, flammable gas and slightly less dense than air, consisting of one carbon atom and one oxygen atom [89]. Fires, faulty combustion heating systems, exhaust from internal combustion engines, and heating gases are the most common causes of CO exposure. Most of the data on the negative effects of CO comes from studies on CO poisoning. CO is said to have antioxidant, anti-inflammatory, and anti-tumorigenic attributes. Nagasaki et al. [90] reported that CO-loaded red blood cells (CO-RBCs) exert renoprotective effects on cisplatin-induced acute kidney injury (AKI). A closer look revealed that cisplatin treatment reduced cell viability in proximal tubular cells via oxidative stress and inflammation. Several studies have further examined the effects of CO exposure at levels that induce tissue hypoxia, particularly in tissues with high O2 utilization requirements (brain, liver, kidney, heart, small intestine) [91]. The kidney is the site of active transport processes that maintain blood homeostasis and, next to the brain, is the largest contributor to basal metabolic rate due to ATP-dependent transport. CO-induced hypoxia would reduce the oxygen available for ATP production in renal mitochondria, thereby adversely affecting kidney function. Acute renal failure (ARF), secondary to rhabdomyolysis, has been observed in cases of acute CO poisoning [91]. Ni et al. [92] reported that acute carbon monoxide poisoning represents one of the leading causes of toxic exposure globally and is associated with multi-organ dysfunction (MOD), with the kidneys being particularly vulnerable. The molecular mechanisms underlying CO-induced AKI remain poorly understood. CO has been proven to induce renal inflammation and apoptosis through CCL4 (chemokine (C-C motif) ligand 4) upregulation and activation of the PI3K/Akt pathway, thus conveying a potential therapeutic target for mitigating COP-induced AKI (COP-AKI) [92].

Studies on the impact of air-borne CO on kidney disease are rare. Based on the Wei et al. [93] cohort study, the CO poisoning cohort had a 6.15-fold higher risk of developing CKD compared with the non-CO poisoning cohort. The study by Yi et al. [94] demonstrated a significant association between air CO levels and renal function in primary GN, highlighting the importance of environmental factors in immune-mediated kidney disease. Different authors propose several mechanisms by which CO can cause kidney injury and lead to GN [95,96]. The main targets of CO comprise intracellular heme proteins, such as cytochrome c oxidase of the respiratory chain, cytochrome P450-dependent monooxygenases, nicotinamide adenine dinucleotide phosphate (NADPH) oxidases, and NO oxidases [95]. The binding of CO to these proteins can induce conformational changes that alter their biological activity [96]. While exposure to low CO levels is considered not only safe but also protective against ischemic injury due to its vasodilatory action [97], exposure to high CO levels has been shown to inhibit mitochondrial respiration and increase ROS generation [98]. Heme oxygenase-1 (HO-1) activity is also associated with CO production. Rat models have demonstrated that mitochondrial respiration inhibition by CO and increased ROS levels lead to the release of free heme, thereby increasing HO-1. HO-1 metabolizes free heme to produce more endogenous CO, thereby decreasing NO production and causing vasoconstriction of renal vessels [99].

### 4.3. The Effects of Lead

In the general population, several heavy metals/trace elements, including lead (Pb), mercury (Hg), and cadmium (Cd), are associated with a rapid decline in kidney function, leading to impaired kidney function (chronic kidney disease) [100]. Cadmium has been associated with Fanconi syndrome, which is a generalized proximal tubular reabsorptive defect. Accumulation of lead within the proximal tubules may result in hyperuricemia and gout, likely through impaired uric acid secretion and a decline in glomerular filtration rate (GFR). In contrast, cadmium-induced nephrotoxicity is marked by elevated urinary excretion of β2-microglobulin, retinol-binding protein, and α1-microglobulin, reflecting proximal tubular dysfunction. Both entities are characterized by tubulointerstitial disease and fibrosis. Yet only early lead nephropathy is characterized by the presence of proximal tubular nuclear inclusion bodies, resulting from lead binding to the lead-binding protein [101].

#### 4.3.1. Influence of Pb on IgA Nephropathy

A study by Liu et al. [102] concluded that lead is associated with elevated IgAN risk in both single-metal and multiple-metal models and is considered a significant factor in IgAN development. The primary indicator was the nonlinear association between lead and decreased eGFR. Moreover, Pb can cause urinary toxic effects even at low concentrations, which are interrelated with CKD. Its influence was investigated both in vitro and in vivo, with a double-positive relationship [102]. Moreover, epidemiological evidence has shown a link between environmental exposure to Pb and an elevated risk of impaired kidney function, laying the groundwork for this and future studies.

Some studies indicate that Pb might induce nephropathy by activating the Nrf2 pathway, thereby triggering apoptosis and blocking autophagy [103]. Elevated blood Pb concentrations were associated with higher serum interferon-γ (IFN-γ) and lower serum IL-13 levels in children living in areas of elevated environmental Pb exposure, which may affect IgG subclass production by regulating Th1/Th2 cytokines [104].

#### 4.3.2. Influence of Pb on Membranous Nephropathy

According to Cremoni et al. [105], occupational lead exposure was more frequent among patients diagnosed with membranous nephropathy than in the broader French workforce, which may favor PLA2R1 epitope spreading. Creatinine at diagnosis among workers exposed to Pb was 1.11 mg/dL, compared with 1.03 mg/dL in nonexposed MN patients; the difference was not statistically significant.

### 4.4. The Effects of Cadmium

Excretion of cadmium (Cd) occurs primarily through the kidneys, with a half-life of 20 to 40 years [106]. Plasma proteins transport Cd to hepatocytes, where the metal is stored in complexes with metallothionein (CdMT) [107]. When hepatocytes die, they release CdMT, which is subsequently filtered by the glomerulus and reabsorbed by the tubules. In the proximal tubule, CdMT is being reabsorbed endosomally. Within endosomes, the Cd–metallothionein (CdMT) complex is delivered to lysosomes, where metallothionein (MT) dissociates from cadmium and undergoes degradation. Subsequently, free Cd is released into the cytoplasm and sequestered through binding to newly synthesized MT. In the distal nephron, however, CdMT uptake occurs via the lipocalin-2 receptor [107].

#### The Influence of Cd on IgA Nephropathy

Although the link between Cd metabolism and IgA nephropathy remains unclear, several studies indicate a correlation [107]. A case study presented by Nogué et al. [106] describes a 39-year-old male patient with IgA mesangial glomerulonephritis and a history of both cigarette smoke and environmental exposure to cadmium. Cd is present in all discussed centers: air pollution, cigarette smoking, and e-cigarette use. The kidney is the primary target organ in cases of chronic cadmium exposure. The proteinuria caused by increased Cd exposure is characterized by the presence of low-molecular-weight proteins in urine, such as beta-2-microglobulin, lysozyme, ribonucleases, immunoglobulin light chains, and retinol-binding protein [106].

Accumulation of cadmium in the renal cortex beyond a critical threshold correlates with a greater incidence of renal impairment in chronically exposed adult populations. It has been estimated to be ~200 µg/g wet weight. Moreover, the cadmium exposure cutoff did not appear to reduce proteinuria among chronically exposed workers [106].

In another case of a 47-year-old man with IgA mesangial glomerulonephritis, optical microscopy showed five glomeruli: one was totally sclerotic, and the others showed light segmental hypercellularity. In two glomeruli, segmental, extra-capillary proliferation and centers of interstitial fibrosis with tubular atrophy were detected [108]. Immunofluorescence was positive for C3 protein and IgA, with a mesangial pattern. This signifies a relation between cadmium and the surrounding working environment for many years and could indicate cigarette use, as the patient had smoked for 15 years.

### 4.5. The Effects of Nickel

Most research on nickel (Ni) is based on animal testing. Studies consistently agree that Ni adversely affects the kidneys, presumably through inflammation, oxidative stress, and lipid peroxidation [4]. For example, Ni caused an inflammatory response and increased the expression of TNF-α and IL-6 proteins; activated NF-κB signaling pathway and oxidative stress; and inhibited activity of renal antioxidant enzymes superoxide dismutase and glutathione reductase, which led to lipid oxidative perturbation or perhaps even to autophagy through AMPK and PI3K/Akt/mTOR signaling pathways in ICR mice [109].

#### The Influence of Ni on IgA Nephropathy

Data on Ni and IgA nephropathy began in 1985 with a paper by Strauss et al. [110]. The authors of the study demonstrated that both animal models of nickel exposure and carcinogenesis yield outcomes similar to those observed in a woman with a dental crown made mainly of nickel. These provide strong evidence for nickel-induced IgA nephropathy. The exposure routes for Ni include ingestion, inhalation, and dermal contact [111]. The absorbed Ni is primarily excreted in the urine. It is worth noting that, in the described patient, hematuria and proteinuria resolved 14 months after removal of intrabody nickel [110]. The number of T cells was also speculated to contribute to kidney injury [110].

### 4.6. The Effects of Acrolein

Acrolein (Acr), an α,β-unsaturated, reactive aldehyde [112], enters the human body through food and the respiratory tract [113]. Given the association of IgA nephropathy with respiratory disturbances, acrolein was also investigated in this context. Although it derives from various sources, including fossil fuels, tobacco, and plastics, the total amount of Acr inhaled through cigarettes exceeds that from all other sources combined. It has been linked to endothelial function and atherosclerosis in smokers [112]. It has been estimated that one cigarette contains 80 µg of acrolein, and the concentration of acrolein in the airway surface lining fluid of the lung may reach 450 µg/L during smoking [112]. In general, α,β-unsaturated aldehydes are quickly neutralized through binding to thiol groups in plasma proteins. This interaction leads to the formation of carbonyl carrier adducts, which can subsequently release the aldehydes at distant sites. Increased Acr intake impairs glucose transport in endothelial cells, potentially contributing to the development of diabetes [113]. Moreover, acrolein is a probable source of ROS and contributes to endothelial dysfunction [112]. This is attributable to the fact that α,β-unsaturated aldehydes readily undergo nonenzymatic reactions with glutathione (GSH) and other thiol-containing enzymes, and activate intracellular ROS sources. Acrolein affects cells by inducing covalent DNA-protein bonds and forming Acr-DNA adducts, thereby damaging cell membranes [113]. Acrolein affects cellular physiological processes by targeting physiologically significant amino acid residues, such as sulfhydryls, imidazoles, and amino groups. Also, acrolein increases endothelial ROS production by activating NADPH oxidase, providing a mechanism underlying smoking’s effects on the vasculature. Importantly, pre-existing kidney dysfunction does not affect either Acr metabolism or excretion [113].

### 4.7. The Effects of Formaldehyde

Formaldehyde (FA) is a colorless, highly reactive, flammable gas with a strong, irritating odor, produced predominantly for industrial applications. Its principal use involves the synthesis of other chemicals, particularly formaldehyde-based resins employed as adhesives and binding agents in the manufacture of wood products, pulp and paper, synthetic fibers, plastics, coatings, and textiles. Urea–formaldehyde resins are also widely utilized as insulation materials in construction. Exposure through inhalation may irritate the nasal, oral, and pharyngeal mucosa [114]. In more severe cases, it can lead to respiratory distress and edema of the larynx and lungs. Oral ingestion is associated with gastrointestinal symptoms, including chest or abdominal pain, nausea, vomiting, diarrhea, and bleeding. Additional manifestations may include tachypnea, jaundice, hematuria, and even renal failure [114]. In the study by Ramos et al. [115], the authors analyzed renal function, oxidative stress, and inflammatory responses in rats exposed to varying concentrations of formaldehyde. Studies have shown that FA has toxic effects on the urinary system. Bakar et al. [116] investigated the potential protective role of proanthocyanidins and vitamin E in mitigating formaldehyde (FA)-induced renal injury in a rat model. They showed epithelial damage in the glomeruli and the renal tubular membrane, hypertrophied tubular cells, and pyknotic nuclei in cells of the loop of Henle. Together, these findings indicate that exposure to FA results in variable lesion levels in renal tissues, thereby promoting the release of vasopressor agents, reducing vasodilatation, and increasing ROS and oxidative stress [117]. Formaldehyde may be present in hair-straightening products despite “formaldehyde-free” labeling, and inhalational exposure can induce renal tubular cytotoxicity. A case has been described involving an adolescent who developed severe acute kidney injury requiring renal replacement therapy shortly after using such a product. Renal biopsy revealed acute tubular necrosis, with imaging findings consistent with microcalcifications [118].

#### The Influence of Formaldehyde on Minimal Change Disease

Formaldehyde is a possible causal factor of MCD, as it has been shown to cause glomerular injury. It can directly interact with cellular components by binding to nucleic acids and proteins, forming complexes [119]. It also contributes to ROS formation, which may subsequently lead to oxidative damage to lipids, proteins, and DNA, thereby exacerbating the effects of primary aldehyde interactions. Mulderrig et al. [120], in their genomic study, found that FA exposure leads to polymerase II stalling, activation of Transcription-Coupled Nucleotide Excision Repair (TC-NER), and polymerase II degradation, perhaps by causing intra-strand DNA crosslinks [121]. This damage can be reversed by TC-NER [120]. In the kidney, formaldehyde has been shown to exert direct cytotoxic effects, leading to acute tubular necrosis, and may additionally provoke immune-mediated mechanisms contributing to AKI [121]. It should be noted that FA might also affect glomeruli indirectly. There are studies on its influence on blood pressure, where, in the longer axis, it affects the glomeruli pathophysiologically [122].

What is more, formaldehyde may be absorbed not only through inhalation but also through the skin and eyes and is eliminated through the urine [121]. This differs from other elaborate substances, as the rest are absorbed only through the lungs. For example, high levels of formaldehyde in beauty salon air and in specimens of hairstylists’ skin can then enter human bodies [121].

**Figure 2:**
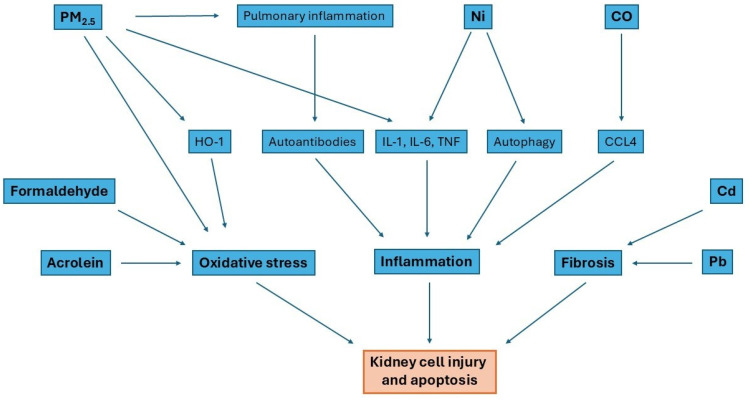
Generalized cascade of mechanisms activated by gases that cause kidney injury.

Figure 2 shows three basic mechanisms by which gases cause kidney injury, which can lead to GN. These are oxidative stress induced by several different factors, inflammation mainly caused by PM_2.5_ and Ni, and fibrosis mainly caused by Pb and Cd.

## 5. Conclusions

Environmental exposure to components of tobacco smoke, e-cigarette aerosols, and ambient air pollution is increasingly recognized as a potential contributor to the development and progression of glomerular diseases. Evidence from epidemiological studies, experimental models, and mechanistic investigations suggests that particulate matter (PM_2.5_, PM_10_), heavy metals such as lead and cadmium, and reactive aldehydes, including acrolein and formaldehyde, may promote glomerular injury through oxidative stress, immune dysregulation, endothelial dysfunction, and direct toxicity to podocytes and mesangial cells.

Although associations between air pollution and CKD progression are better established, data specifically addressing primary glomerulonephritis remain limited and heterogeneous. The current literature does not yet provide definitive mechanistic pathways linking individual pollutants to distinct GN phenotypes. Moreover, variability in exposure assessment, regional environmental profiles, and population susceptibility complicates causal interpretation.

Importantly, patients with pre-existing CKD appear to be more vulnerable to the harmful effects of air pollutants, suggesting that environmental exposure may act both as a trigger and as a disease-modifying factor. This has significant public health implications, particularly in industrialized and densely populated regions.

Future research should focus on mechanistic studies examining pollutant-induced injury in glomerular structural cells, longitudinal population-based analyses stratified by GN subtype, and region-specific assessments of environmental risk.

A clearer understanding of the interaction between environmental toxicants and immune-mediated kidney injury may facilitate the development of preventive strategies and inform public health policies to reduce exposure-related renal risk.

## Data Availability

No new data were created.

## Abbreviations

The following abbreviations are used in this manuscript:

Acr	Acrolein
AKI	Acute kidney injury
ARF	Acute renal failure
Cd	Cadmium
CKD	Chronic kidney disease
CO	Carbon monoxide
CO_2_	Carbon dioxide
EEA	European Environment Agency
ESRD	End-stage renal disease
FSGS	Focal segmental glomerulosclerosis
ETS	Environmental tobacco smoke
EU	European Union
FA	Formaldehyde
GN	Glomerulonephritis
GFR	Glomerular filtration rate
HOX	Heme oxygenase-1
IgAN	IgA nephropathy
MM	Membranous nephropathy
MCD	Minimal change disease
MSS	Mainstream smoke
Ni	Nickel
NO	Nitric oxide
PAHs	Polycyclic aromatic hydrocarbons
Pb	Lead
PLA2R	Phospholipase A2 receptor
PM	Particulate matter
RNS	Reactive nitrogen species
ROS	Reactive oxygen species
SSS	Sidestream smoke
TSNAs	Tobacco-specific nitrosamines
VOC	Volatile organic compounds
WHO	World Health Organization
